# Hidden in morphology, revealed by molecular genetics: synonymization of *Gogatea burmanicus* (Chatterji, 1940) with *Gogatea serpentum* (Gogate, 1932) (Digenea: Cyathocotylidae)

**DOI:** 10.1017/S0031182025101054

**Published:** 2026-04

**Authors:** Sila Viriyautsahakul, Vachirapong Charoennitiwat, Kittipong Chaisiri, Abigail Hui En Chan, Chonlada Tippawan, Alexis Ribas, Panithi Laoungbua, Tanapong Tawan, Urusa Thaenkham, Napat Ratnarathorn

**Affiliations:** 1 Applied Animal Science Laboratory, Department of Biology, Faculty of Science, Mahidol University, Bangkok, Thailand; 2 Laboratory of Helminth Biodiversity and Drug Development, Department of Helminthology, Faculty of Tropical Medicine, Mahidol University, Bangkok, Thailand; 3 Parasitology Section, Department of Biology, Healthcare, and Environment, Faculty of Pharmacy and Food Science, University of Barcelona, Barcelona, Spain; 4 Institute of Research in Biodiversity (IRBio), University of Barcelona, Barcelona, Spain; 5 Snake Farm, Queen Saovabha Memorial Institute, The Thai Red Cross Society, Bangkok, Thailand

**Keywords:** *Enhydris enhydris*, *Gogatea*, gonad, molecular genetics, morphology, synonym

## Abstract

The classification of the digenean genus *Gogatea* Lutz, 1935 has been complicated for almost a century due to morphological variability and reliance on limited diagnostic traits. This study re-evaluates the taxonomic status of *Gogatea serpentum* (Gogate, 1932) and *Gogatea burmanicus* (Chatterji, 1940) using an integrative framework combining morphology and molecular phylogenetics. Trematodes were recovered from the gallbladder and intestine of the rainbow water snake (*Enhydris enhydris*) in southern Thailand. Morphological investigations included morphometrics, acetocarmine-stained preparations, scanning electron microscopy and multivariate analyses, while molecular analyses used mitochondrial COI and nuclear ITS2 and 28S rRNA markers. Both gonad-bearing and gonad-less individuals exhibited identical sequences across all markers, forming a strongly supported monophyletic group. Morphological variation was restricted to the presence or absence of gonads, with no separation detected by principal component analysis. These findings support the synonymization of *G. burmanicus* as a junior synonym of *G. serpentum* (following the original spelling by Gogate, 1932, as validated under the ICZN [International Code of Zoological Nomenclature]). The occurrence of gonad-less adults represents a biologically intriguing phenomenon, the causes of which remain unresolved but may involve developmental, host-related, or ecological factors. This study underscores the importance of combining molecular and morphological approaches for accurate delimitation of morphologically plastic digeneans. Updated morphological descriptions and molecular data for *G. serpentum* are provided, including morphometrics, staining profiles, scanning electron microscopy micrographs and genetic sequences. These findings refine the taxonomy of *Gogatea*, advance knowledge of helminth diversity in semi-aquatic snakes and support broader efforts in parasite systematics, host–parasite ecology and biodiversity monitoring in Southeast Asia.

## Introduction

The genus *Gogatea* Lutz, 1935, belonging to the family Cyathocotylidae, currently comprises nine recognized species: *Gogatea joyeuxi* (Hughes, 1929), *Gogatea serpentum* (Gogate, 1932), *G. burmanicus* (Chatterji, 1940), *Gogatea karachiensis* Farooq, 1973, *Gogatea taiwanensis* (Fischthal & Kuntz, 1975, *Gogatea mehri* Mehra, 1947, *Gogatea bijirrii* Achatz, 2024, *Gogatea acrochordi* Achatz, 2024 and *Gogatea anacetabulata* Achatz, 2024 (Achatz et al., 2024). These digenean trematodes are distributed across South Asia, Southeast Asia and Australia and are known to parasitize reptiles, particularly snakes. Members of this genus are characterized by an elongate, linguiform body with a distinctly extended posterior region housing the posterior testis. A large, oval holdfast organ with vitelline follicles confined within it is also diagnostic. These features clearly differentiate *Gogatea* from other genera (Lutz, 1935; Jones et al., 2005; Achatz et al., 2024).

In Southeast Asia, various semi-aquatic snakes have been reported as hosts of *Gogatea* species. For instance, *G. serpentum* has been documented in the intestine of the checkered keelback snake, *Fowlea piscator* (Schneider, 1799) and the gallbladder of the tentacle snake, *Erpeton tentaculatum* Lacépède, 1800 (Dubois, 1938; Mehra, 1947; Singh, 1956; Simha, 1958; Frank, 1966; Gupta, 1967), while *G. burmanicus* has been found in the intestine of the rainbow water snake, *Enhydris enhydris* (Schneider, 1799) (Chatterji, 1940).

In addition to generic characteristics, *G. serpentum*, the type species of the genus, is characterized by a flattened anterior body with a cylindrical posterior region, a well-developed oral sucker and cirrus sac, a prominent holdfast organ overlapping the ventral sucker, tandem testes and a vitellarium composed of large follicles confined to the holdfast organ (Jones et al., 2005).

In contrast, *G. burmanicus* shares many of *G. serpentum*’s morphological characteristics but is distinguished by the apparent absence of gonads in specimens that otherwise appear sexually mature, along with the presence of a larger, muscular cirrus sac (Mehra, 1947; Jones et al., 2005). Despite these differences, both species were described from the same host region and infection site – specifically, the intestines of semi-aquatic snakes – based solely on morphology (*sensu* Chatterji, 1940; Mehra, 1947), at a time when molecular tools were not yet available. However, reliance on gonadal visibility as a primary diagnostic character may be problematic, particularly given known variability in reproductive development (Schemmel and Brown-Peterson, 2023), post-mortem preservation effects (Bryant and Boekelheide, 2007), or intraspecific competition (Saldanha et al., 2009; Viriyautsahakul et al., 2025). The dependence on a single morphological trait has raised concerns regarding the robustness of the original species distinction (Cribb et al., 2025). Cases like this underscore the broader challenge of species delimitation in digeneans, which can be obscured by morphological plasticity, developmental stages, or host-induced variation (Cribb et al., 2025; Rojas et al., 2025). The application of integrative taxonomy – combining molecular, morphological, geographical and host data – has become increasingly important in resolving such ambiguities among parasitic taxa (Rojas et al., 2025).

During our investigation of helminth parasites in snakes, specimens of *E. enhydris* collected from southern Thailand were occasionally found to harbour digeneans in their digestive organs. Initial morphological examination identified these as *G. serpentum* and *G. burmanicus*, differing only in the presence or absence of gonads. Subsequent molecular genetic analyses revealed identical sequences between the 2 taxa, challenging their status as separate species and raising the possibility that they represent morphologically variable forms of a single species. This observation prompted a re-evaluation of the taxonomic status of *G. burmanicus*. Clarifying the taxonomic identity of these trematodes is essential not only for systematic accuracy but also for improving our understanding of host specificity, parasite diversity and the biogeography of helminths in Southeast Asian ecosystems (Poulin et al., 2019a). Such efforts contribute to broader initiatives in biodiversity monitoring and parasite conservation (Poulin, 2014).

In this study, *Gogatea* specimens with and without gonads, collected from *E. enhydris*, were examined using an integrative approach combining morphological analysis and molecular phylogenetics. Morphological investigations included general morphological examinations, colour-stained preparations, scanning electron microscopy and multivariate analysis to assess character variation. Phylogenetic relationships were inferred using 3 genetic markers: the mitochondrial cytochrome c oxidase subunit I (*COI*), the large subunit of nuclear ribosomal RNA (28S rRNA) and the ribosomal internal transcribed spacer 2 (ITS2). The aims of this study are (1) to demonstrate that *G. burmanicus* (Chatterji, 1940) syn. n. is conspecific with *G. serpentum* (Gogate, 1932), and should therefore be synonymized; and (2) to evaluate the extent of morphological variation within the genus *Gogatea*, highlighting the importance of integrative taxonomy in resolving cases of morphologically plastic species, particularly among parasitic helminths.

## Materials and methods

### Host and parasite specimen preparation

Rainbow water snakes (*E. enhydris*) were sampled from Nakhon Si Thammarat and neighbouring provinces in southern Thailand, with the assistance of local villagers and wildlife rescuers. The collected specimens were subsequently transported to the Snake Farm at the Queen Saovabha Memorial Institute in Bangkok. Between 2021 and 2025, a total of 119 snakes that died during quarantine at the facility were dissected, and their remains were transferred to the Department of Helminthology, Faculty of Tropical Medicine, Mahidol University, Bangkok, for parasitological examination. Prior to dissection, the snakes were stored at −20 °C.

Before dissection, each specimen underwent external morphological examination and measurements following the criteria outlined by Cox et al. (2012) to confirm species identity and record host information. Dissections were conducted using the methods described by Ratnarathorn and Kongrit (2025). As the gallbladder was the primary site of infection, it was carefully removed, placed in Petri dishes with water to prevent desiccation and subsequently opened and examined under a stereomicroscope (Olympus SZ51, Japan) for the presence of *Gogatea* spp. When detected, parasites were gently isolated and transferred to Petri dishes for further examination.

Among the recovered trematodes, 2 distinct morphological groups were identified: (1) individuals possessing fully developed gonads and (2) individuals lacking gonads. Four well-preserved specimens with developed gonads were fixed in 2.5% glutaraldehyde in 0.1 m sucrose phosphate buffer for scanning electron microscopy. The remaining parasites were preserved in 70% ethanol. In addition, 6 immature trematodes were recovered from the small intestine of 1 snake; they were preserved in 70% ethanol for subsequent molecular analysis to verify their conspecificity with the specimens derived from the gallbladder.

### Morphological study

**Figure 1.**
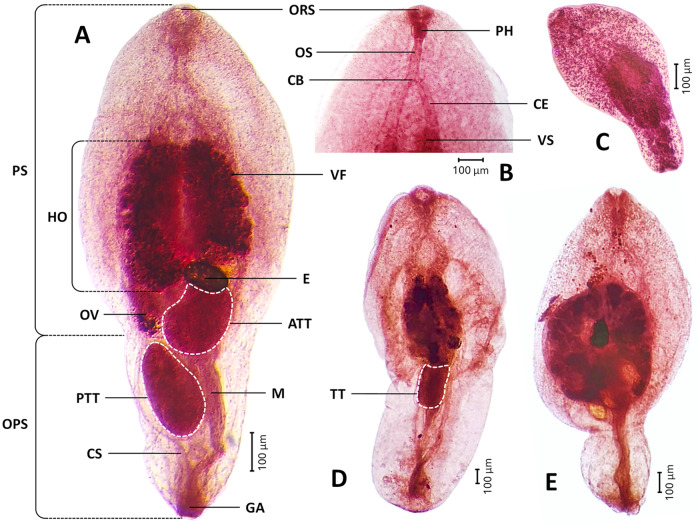
Permanent slides (acetocarmine dye) of *Gogatea serpentum*: (A) entire body of a mature specimen with complete-gonad (white-dashed lines show 2 testes), ventral view; (B) anterior region, ventral view; (C) entire body of an immature specimen, ventral view; (D) entire body of a mature specimen with incomplete gonad (white-dashed line shows 1 testis), ventral view; and (E) entire body of a mature specimen without gonad, ventral view. Abbreviations: ATT, anterior testis; CB, caeca bifurcation; CE, caecum; CS, cirrus sac; E, eggs; GA, genital atrium; HO, holdfast organ; M, metraterm; OPS, ophistosoma; ORS, oral sucker; OS, oesophagus; OV, ovary; PH, pharynx; PTT, posterior testis; PS, prosoma; TT, testis; VF, vitelline follicle; VS, ventral sucker. Figure 1 long description.

For the specimens with gonads – which were the majority of the parasites found – 18 complete individuals from 4 out of 25 infected snakes were selected from the 70% ethanol stock for morphological study and permanent slide preparation. The remaining specimens were unsuitable for measurement due to deformation or incompleteness, with a few retained for genetic analysis. An additional 3 individuals without gonads were also prepared for permanent slide mounting. Each selected specimen was fixed in 10% formalin overnight, rinsed in distilled water for 3 min and stained with acetocarmine for 48 h. Destaining was performed using a 1% HCl solution for 10 s, followed by a graded ethanol dehydration series (70%, 80%, 90%, 95% and 100%), with each step lasting 12 min – except for the 95% ethanol and absolute ethanol steps, which lasted 20 min and 1 h, respectively, to ensure complete dehydration. The specimens were then immersed in a 1:1 solution of 100% ethanol and xylene for 5 min, briefly placed in pure xylene and subsequently mounted in Permount™ on glass slides with coverslips. The mounted slides were left at room temperature for several days to ensure complete drying, and later photographed using the same light microscope (see Figure 1 for illustrations).

**Figure 2.**
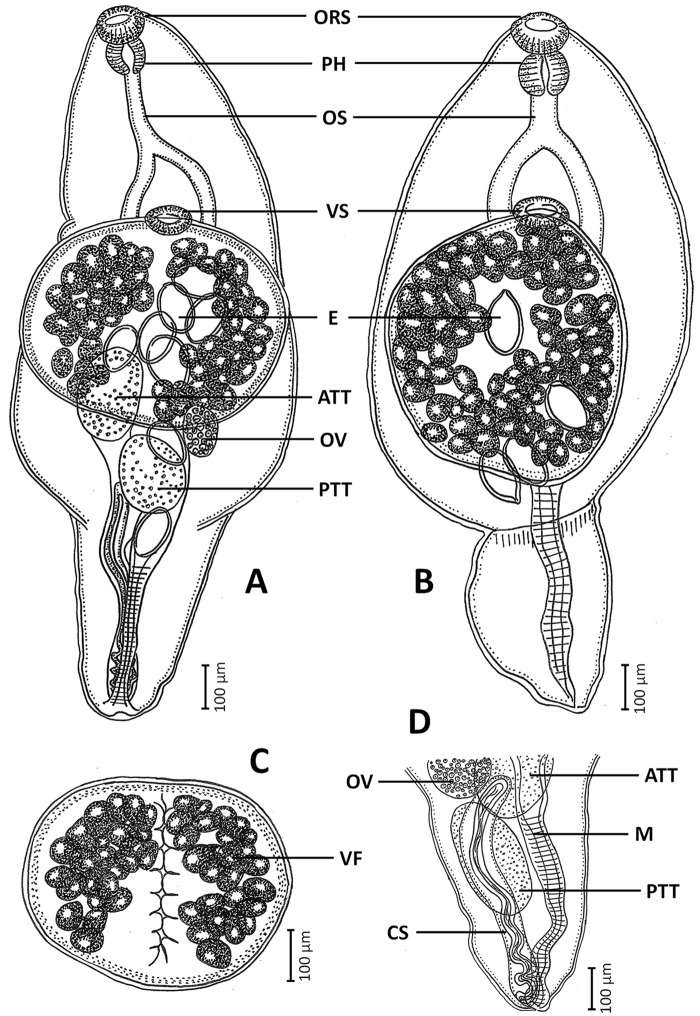
Camera lucida drawing illustrations of *Gogatea serpentum*: (A) entire body of a mature specimen with complete gonads, ventral view; (B) entire body of a mature specimen lacking gonads, ventral view; (C) holdfast organ, ventral view; and (D) posterior region of a mature specimen with complete gonads, ventral view. Abbreviations: ATT, anterior testis; CS, cirrus sac; E, eggs; M, metraterm; ORS, oral sucker; OS, oesophagus; OV, ovary; PH, pharynx; PTT, posterior testis; VF, vitelline follicle; VS, ventral sucker. Figure 2 long description.

The morphological characters of the mounted specimens were measured using ZEN 2 Blue Edition software connected to an inverted microscope (Zeiss Primovert, Germany) equipped with a Zeiss Axiocam, while the illustrations (Figure 2) were created using a light microscope with a camera lucida (Leitz, Wetzlar, Germany). A total of 23 morphological characters were recorded, including body length, forebody length (i.e. distance from the oral sucker to the ventral sucker), prosoma length, prosoma width (i.e. maximum body width), pharynx length, pharynx width, oesophagus length, oral sucker length, oral sucker width, ventral sucker length, ventral sucker width, holdfast organ length, holdfast organ width, cirrus sac length, cirrus sac width, ovary length, ovary width, length and width of each testis, egg length and egg width (Table 1; Supplementary Tables S1–S3). Three of these characters – forebody length, oesophagus length and cirrus sac length – were also calculated as percentages of the total body length. All measurements were expressed in micrometres (μm) (Table 1).

**Table 1.** Morphological features and measurement data of *Gogatea serpentum* examined in this study (including both gonad-bearing and gonad-less specimens), compared with those from previous studies and *Gogatea burmanicus* syn. n. All measurements are in micrometres (µm) and are presented as ranges with the mean ± standard deviation in parentheses Table 1 long description.

Parasite species	*Gogatea serpentum*				*Gogatea burmanicus*
Reference	This study		Gogate (1932), Dubois (1938), Mehra (1947), Frank (1966) and Gupta (1967)		Chatterji (1940)
Host species	*Enhydris enhydris*		*Fowlea piscator*	*Erpeton tentaculatum*	*Enhydris enhydris*
Site of infection	Gallbladder		Intestine	Gallbladder	Intestine
Locality	Nakhon Si Thammarat, Thailand		Myanmar	Bangkok, Thailand	Rangoon, Myanmar
Specimen number	18 (*with gonad*)	3 (*without gonad*)	1−8	4	10
Body length	1258−2087 (1643 ± 259)	1356−1723 (1558 ± 186)	590−1440	2630−2760	1148−1600
Forebody length	316–748 (498 ± 118)	356–536 (457 ± 92)	—	—	—
Prosoma length	729–1432 (1115 ± 209)	935–1362 (1163 ± 215)	770	—	630–900
Prosoma width	494–965 (683 ± 136)	494–1366 (876 ± 446)	380–650	980–1080	250–270
Opisthosoma length	291–705 (510 ± 117)	388–418 (400 ± 16)	—	—	120–250
Opisthosoma width	215–531 (356 ± 104)	218–347 (302 ± 73)	—	—	120–168
Prosoma: opisthosoma length ratio	1.5–3.9 (2.2 ± 0.6)	2.3–3.3 (2.9 ± 0.5)	—	—	—
% Forebody length	22–45 (30 ± 6)	26–31 (29 ± 3)	—	—	—
Oral sucker length	59–143 (88 ± 24)	72–124 (99 ± 26)	62–117	138	63
Oral sucker width	82–154 (117 ± 19)	90–145 (119 ± 28)	76–138	190	75
Ventral sucker length	51–109 (70 ± 23)	49–82 (65 ± 17)	30–64	95–100	30–33
Ventral sucker width	39–136 (83 ± 27)	85–105 (98 ± 11)	35–49	100–105	33–39
Ratio sucker’s length	1–2 (1)	1–2 (2)	—	—	—
Ratio sucker’s width	1–3 (2 ± 1)	1 (1)	—	—	—
Holdfast organ length	395–747 (518 ± 107)	406–579 (489 ± 87)	190–440	—	135–210
Holdfast organ width	350–812 (486 ± 146)	447–602 (532 ± 78)	140–300	—	120–170
Pharynx length	57–85 (70 ± 9)	49–80 (66 ± 16)	30–50	105	36–42
Pharynx width	56–101 (74 ± 12)	43–84 (70 ± 23)	30–53	100	30–36
Oesophagus length	82–212 (135 ± 41)	86–171 (128 ± 42)	19–106	—	87–102
% Oesophagus length	6–12 (8 ± 2)	6–10 (8 ± 2)	—	—	—
Anterior testis length	165–263 (218 ± 29)	Absent	112–166	320–345	Absent
Anterior testis width	128–211 (160 ± 25)	Absent	108–166	190	Absent
Posterior testis length	148–342 (234 ± 45)	Absent	99–190	350	Absent
Posterior testis width	127–259 (170 ± 34)	Absent	75–166	190	Absent
Cirrus sac length	343–827 (593 ± 120)	518–660 (594 ± 72)	300–473	750–800	240–390
Cirrus sac width	57–126 (86 ± 17)	77–118 (102 ± 22)	48–60	75–95	60–80
% Cirrus sac length	27–45 (36 ± 6)	32–45 (38 ± 6)	—	—	—
Ovary length	79–130 (107 ± 15)	Absent	78–140	95–100	Absent
Ovary width	51–111 (75 ± 16)	Absent	66–120	100–105	Absent
Number of eggs	0–15 (6 ± 5)	3–5 (4 ± 1)	1–2	6–7	1–3
Egg length average	119–151 (135 ± 9)	130–150 (141 ± 10)	60–153	150–164	99–129
Egg width average	69–100 (86 ± 10)	79–95 (89 ± 9)	30–90	92–96	72–84

**Figure 3.**
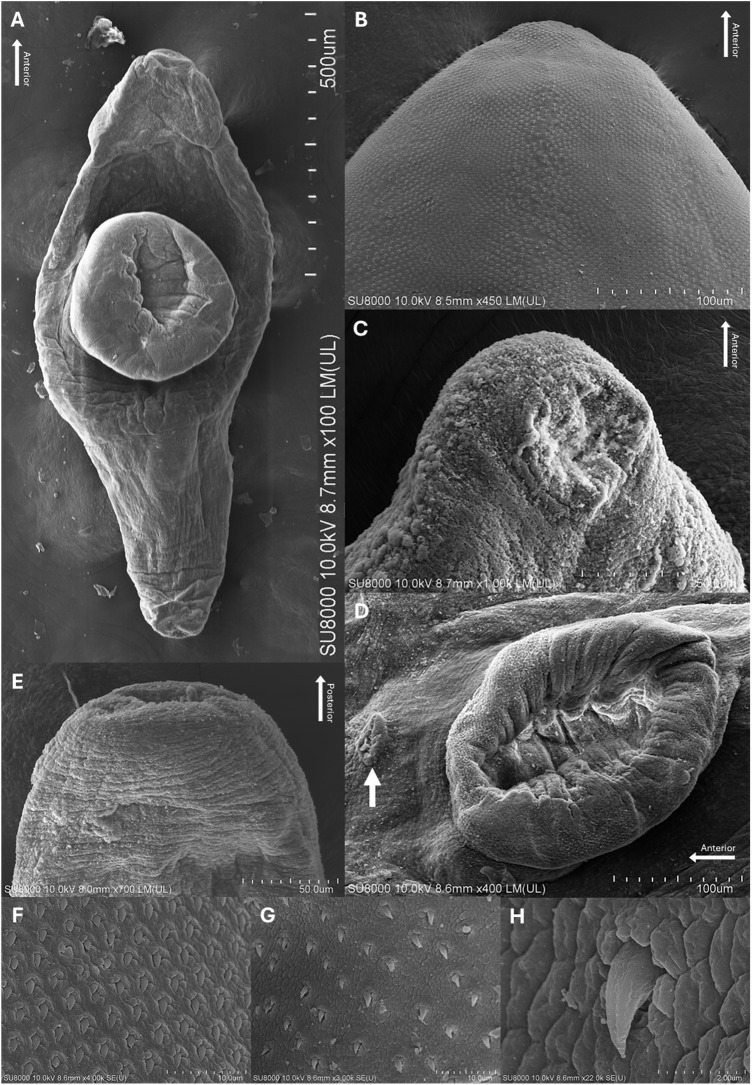
Scanning electron micrographs of *Gogatea serpentum*: (A) entire body with a very large protruded holdfast organ, ventral view; (B) anterior region with densely distributed dot-like tegumental spines, dorsal view; (C) oral sucker, anterior region, ventral view; (D) large protruded holdfast organ with small ventral sucker anteriorly (white arrow), ventral view; (E) genital pore at apical end, ventral view; (F) densely distributed tegumental spines, anterior body; (G) sparsely distributed tegumental spines, posterior body; and (H) magnified view of tegumental spine. Figure 3 long description.

Scanning electron microscopy (SEM) analysis was conducted on 4 specimens that were initially preserved and fixed in 2.5% glutaraldehyde solution to assess tegument spine characteristics, including shape, size and distribution pattern. Specimen preparation was carried out at the Department of Tropical Pathology, Faculty of Tropical Medicine, Mahidol University. Each specimen underwent secondary fixation in 1% osmium tetroxide in 0.1 m SPB, followed by ethanol dehydration and drying using a critical point dryer (CPD300 auto, Leica, Wetzlar, Germany). The dehydrated specimens were then coated with gold using a sputter coater (Q150R PLUS, Quorum, East Sussex, UK). The samples were subsequently transferred to the Central Instrument Facility, Faculty of Science (Phaya Thai Campus), Mahidol University, where SEM imaging was performed (Hitachi SU8010, Hitachi High-Tech, Japan) (see Figure 3 for illustrations).

**Figure 4.**
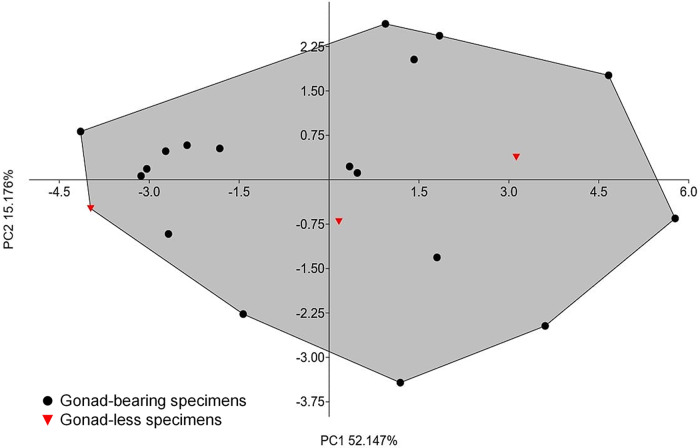
Principal component analysis (PCA) of *Gogatea serpentum* was performed using 16 morphological characters, accounting for 67.323% of the total variance. Black dots and red triangles represent specimens with and without gonads, respectively. Figure 4 long description.

Principal component analysis (PCA) was conducted to analyse the multivariate data matrix of 16 morphometric characters using PAST version 4.06b (Hammer et al., 2001). To illustrate morphological variation among the specimens (Figure 4), the correlation model was used to generate 2-dimensional scatter plots with the corresponding percentage variances. PCA was performed on all 21 mounted individuals, including the 3 parasites lacking gonads. Ventral sucker, ovary and egg measurements were excluded from the analysis due to being unobservable in some specimens; likewise, all gonad-related measurements were omitted, as these structures were absent in the gonad-less individuals (Supplementary Tables S2 and S3).

### Molecular and phylogenetic study

The molecular analysis was performed to compare whether the individual group with gonads and without gonads are conspecific. DNA was extracted from 2 specimens of individuals with gonads, and one parasite from the individuals without gonads using the Genomic DNA Mini Kit (Geneaid Biotech Ltd., New Taipei City, Taiwan) according to the manufacturer’s protocol. Additionally, the DNA was also extracted from 2 immature parasites found in the small intestine. The DNA amplification was conducted on 3 regions, including the nuclear 28S ribosomal RNA (28S rRNA), the nuclear ITS2 and the mitochondrial cytochrome *COI*. The selected genes are those that are widely utilized in molecular identification and genetic diversity studies in trematodes (Thaenkham et al., 2022). The obtained sequences were subsequently concatenated using the aligned sequences from all 3 genetic markers. These targeted genes were amplified by polymerase chain reaction (PCR) using a T100™ thermocycler (Bio-Rad) in a 30 µL mixture containing 15 µL of 2× i-Taq master mix (iNtRON Biotechnology, Gyeonggi, South Korea), 10 µm of each primer and 1 ng µL^−1^ of DNA. The PCR details for each of the targeted regions including the primers used, expected amplicon lengths and the thermocycling profile are provided in Supplementary Table S4 (Curran et al., 2011; Routtu et al., 2014; Van Steenkiste et al., 2015; Besprozvannykh et al., 2018). The PCR amplicons were visualized on a 1% agarose gel stained with SYBR Safe (Thermo Fisher Scientific, USA). The products that were successfully amplified, as indicated by clear bands on the agarose gel, were sent for sequencing using Fast Next-Generation Sequencing (Tsingke, Beijing, China). The obtained nucleotide sequences were submitted to the NCBI database under the accession numbers PV988016–18 for 28S rRNA, PV988019–21 for *COI* and PV988350–52 for ITS2.

**Figure 5.**
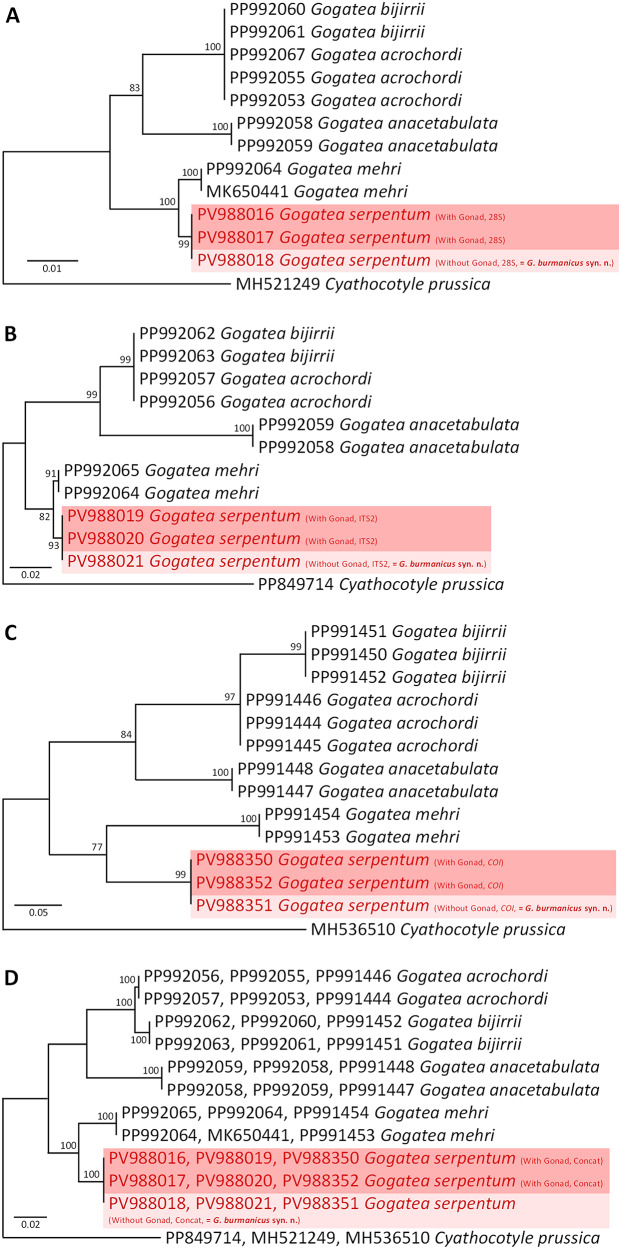
Phylogenetic analyses were conducted using the maximum likelihood method in MEGA-12 based on the 28S rRNA (A), ITS2 (B), *COI* (C), and concatenated sequences of all 3 markers (D) from available species within the genus *Gogatea*. Branch length scale bars indicate the number of substitutions per site, and node values represent bootstrap support. Specimens of *Gogatea serpentum* with and without gonads (the latter corresponding to *Gogatea burmanicus* syn. n.) Identified in this study are highlighted in dark red and light red font/boxes, respectively. Figure 5 long description.

After sequencing, the partial sequences of each marker were manually inspected and edited using BioEdit version 7.2.5. Multiple sequence alignment was performed by ClustalX 2.1 with reference sequences of other *Gogatea* species. Phylogenetic analysis was conducted using maximum likelihood (ML) in MEGA-11. Best-fit nucleotide substitution models were determined for each marker: the Hasegawa–Kishino–Yano model with an additional parameter accounting for a proportion of invariable sites (+I) for 28S rRNA and *COI*, the Jukes–Cantor with an additional parameter accounting for a proportion of invariable sites (+I) for ITS2 and the general time reversible model with a discrete gamma distribution (+G) for concatenated sequences from all 3 markers. Phylogenetic trees were constructed using 1000 bootstrap replicates to ensure analytical robustness (Hall, 1999; Thompson et al., 2002; Tamura et al., 2013). The *COI*, 28S rRNA and ITS2 analyses included sequences from all available *Gogatea* species, with *Cyathocotyle prussica* used as the outgroup for each marker. All sequences were obtained from GenBank (Figure 5; Supplementary Table S5).

## Result

### Morphological remarks

The trematode *G. serpentum* (Gogate, 1932) was found infecting 25 of 119 examined *E. enhydris*, indicating a moderate prevalence of 21.0%, with intensities ranging from 1 to 33 parasites per snake (average of 5 parasites per infected snake). In total, 131 parasites were recovered from the gallbladder of the snakes. Of these, 14 parasites recovered from 3 different infected snakes (with intensities of 2, 4 and 8) lacked gonads, while the rest had both ovaries and testes present. Additionally, 6 immature parasites – identified by their small size and the absence of a visible uterus and eggs – were collected from the small intestine of one of the infected snakes.

Morphological identification confirmed that the parasites obtained from *E. enhydris* in this study are *G. serpentum*. The specimens possess a large holdfast organ (i.e. adhesive apparatus) with a vitellarium consisting of large vitelline follicles confined within it, which is a key characteristic of the genus *Gogatea*. The morphometric measurements of the species are mostly similar in size to those reported from other populations of *G. serpentum* previously recorded in the Southeast Asian region (Table 1; Supplementary Table S1). The presence of a small ventral sucker rules out *G. anacetabulata* (Achatz et al., 2024), which is the only other species reported from Southeast Asia but lacks a ventral sucker.

Based on 18 parasitic adult specimens with complete gonads, the description of the parasite is as follows (Table 1; Supplementary Tables S1 and S2) (all measurements in μm): body elongated, linguiform, with a total length of 1258–2087 (Figures 1A, 2A and 3A). Forebody (oral to ventral sucker) 316–748 (Figure 1B). Prosoma oval (Figure 1A), 729–1432 × 494–965. Opisthosoma tail-like, elongated, cylindrical, 291–705 × 215–531. Oral sucker well-developed, 59–143 × 82–154 (Figures 2A,B and 3C). Ventral sucker small, round, overlapping with a large holdfast organ, 51–109 × 39–136 (Figures 2A,B and 3D). Ratio of ventral sucker to oral sucker: length 1:1, width 2:1. Holdfast organ well-developed, very large, round, or oval with longitudinal opening, 395–747 × 350–812 (Figures 1A, 2C and 3D). Prepharynx short or absent. Pharynx small, 57–85 × 56–101. Oesophagus short, 82–212 (E = 8%) (Figures 1B and 2A,B). Tegumental spines minute, sharply pointed; most numerous and densely arranged on forebody, progressively sparser towards posterior end (Figure 3B, F–H).

Testes round, tandem, situated posterior to ventral sucker (Figures 1A and 2A,D). Anterior testis 165–263 × 128–211; posterior testis slightly larger, 148–342 × 127–259. Cirrus sac elongate, narrow, 343–827 × 57–126, extending from level of anterior testis to genital pore (CS = 36%). Metraterm thick, joined with cirrus sac at genital atrium (Figures 1A and 2D). Genital pore terminal (Figure 3E). Ovary round or oval, intertesticular or at the same level as anterior testis (Figure 2A,D). Vitellarium composed of large follicles confined to holdfast organ (Figure 2C). Eggs oval, large, 119–151 × 69–100, with 0–15 eggs per individual.

### Morphological variation

The morphology of 3 other individuals without gonads showed measurements similar to those with gonads and an identical overall appearance, except for the absence of testes and ovary, and a slightly thicker wall of the cirrus sac, which fits well with the description of *G. burmanicus* (Chatterji, 1940) (see full measurements in Table 1; Supplementary Tables S1 and S3; Figures 1E and 2B). Interestingly, there was also one individual who had only one testis present (Figure 1D). In addition, the individuals from the small intestine appeared to be immature or underdeveloped, as indicated by their noticeably smaller size (700–900 μm) and the lack of developed internal organs (Figure 1C).

PCA conducted on both complete-gonad (*n* = 18) and incomplete-gonad (*n* = 3) individuals revealed no distinct separation or morphological clustering, as shown in the 2-dimensional PC1–PC2 plots (Figure 4). The analysis accounted for 67.323% of the total variance, supporting the morphological homogeneity of the *G. serpentum* samples. Interestingly, the 3 gonad-less individuals did not cluster together but were instead dispersed throughout the plot, further indicating no consistent morphological difference between gonadal and non-gonadal specimens. Based on these findings, the synonymization of *G. burmanicus* as a new synonym of *G. serpentum* is proposed.

### Genetic characterization and phylogenetic position

Despite exhibiting different morphologies, phylogenetic analyses using the 3 markers (ITS2, 28S rRNA and *COI*) showed that all specimens were 100% identical, with no genetic variation observed (Figure 5A–C). Molecular evidence thus further reinforces morphometric analyses, supporting our proposal that the *Gogatea* specimens, which were initially thought to be *G. burmanicus* due to the lack of gonads, are identical to *G. serpentum*. All 4 phylogenies revealed that *G. serpentum* is genetically closest to *G. mehri*, forming a monophyletic clade. The genetic distance obtained between these 2 species were 0.7% for both 28S rRNA and ITS2 markers, and 14.2% for *COI. Gogatea bijirrii, G. acrochordi* and *G. anacetabulata* formed another monophyletic clade, with *G. bijirrii* and *G. acrochordi* sharing a closer genetic relationship than *G. anacetabulata*. Additionally, *G. bijirrii* and *G. acrochordi*, recently described from Australia, were genetically indistinguishable in both the nuclear 28S rRNA and ITS2 analyses (Figure 5A,B), while their *COI* sequences were clearly distinct, differentiating them as separate species (Figure 5C).

### Nomenclatural note

The species was originally described as *Prohemistomum serpentum* by Gogate (1932). Subsequent authors have variably referred to it as *Gogatea serpentium* or *Gogatea serpantium* (Lutz, 1935; Frank, 1966; Gupta, 1967), resulting in some inconsistency in the literature. However, according to the International Code of Zoological Nomenclature (ICZN, 1999: Articles 32–33.3), the original spelling *serpentum* should be retained unless demonstrably incorrect. As there is no evidence that Gogate’s use of *serpentum* was inadvertent, this spelling is regarded as the correct and valid one. In his monograph on trematodes, Yamaguti (1958) also employs the term *serpentum*, citing the original description by Gogate (1932) and the subsequent work of Lutz (1935), and thereby retained the original nomenclature. In addition, recent taxonomic consensus (Chatterji, 1940; Achatz et al., 2024) also follows *serpentum* in accordance with the original description, further supporting its validity. The alternative spellings *serpentium* and *serpantium* are considered incorrect subsequent spellings under ICZN Article 33.3 and are treated here as such.

***Taxonomic summary***

Phylum: Platyhelminthes Minot, 1876

Class: Trematoda Rudolphi, 1808

Order: Plagiorchiida La Rue, 1957

Family: Cyathocotylidae Mühling, 1896

Genus: *Gogatea* Lutz, 1935

**Species**: *Gogatea serpentum* (Gogate, 1932)

**Host**: *Enhydris enhydris* (Schneider, 1799)

**Locality**: Aquatic areas such as lakes, ponds and swamps in Nakhon Si Thammarat (e.g. Pak Phraek [8°14′56.04″ N, 100°13′18.96″ E], Thung Song [8°17′18.6″ N, 99°29′42.6″ E] District) and neighbouring provinces in southern Thailand (e.g. Songkhla Lake [7°12′34.2″ N, 100°27′43.56″E]) served as collection sites. However, the exact coordinates of individual hosts were not documented.

**Collection date**: 1 January 2021 to 7 February 2025

**Site of infection**: gallbladder and small intestine (duodenum)

**Parasite intensity**: 1–33 worms, mean approximately 5

**Parasite prevalence**: 0.21 (25 out of 119 hosts infected)

**Specimens deposited**: Department of Helminthology, Faculty of Tropical Medicine and Department of Biology, Faculty of Science, Mahidol University

## Discussion

The trematode genus *Gogatea* was represented by specimens discovered in the gallbladder and duodenum of the rainbow water snake (*E. enhydris*) in southern Thailand, identified based on their morphology and distribution. This study proposes the synonymization of *G. burmanicus* syn. n. with *G. serpentum*, as some adult parasites lacked gonads, corresponding to the key characteristics originally described for *G. burmanicus* by Chatterji (1940) from 10 specimens recovered from the intestine of *E. enhydris* in Rangoon, Myanmar (Chatterji, 1940; Mehra, 1947). Molecular analyses, however, revealed that gonad-less individuals clustered in a strongly supported monophyletic group with specimens possessing fully developed gonads across multiple gene markers, indicating conspecificity. Accordingly, *G. burmanicus* syn. n. is here recognized as a junior synonym of *G. serpentum*.

The use of molecular data in trematode taxonomy has become increasingly crucial for resolving both inter- and intraspecific morphological variability, as well as for detecting cryptic species (Blasco-Costa et al., 2016). Trematodes show not only intraspecific differences in morphology across different life cycle stages but also variation among individuals within the same stage (Presswell and Bennett, 2020; Viriyautsahakul et al., 2025). This can arise from multiple factors, including differences in host species, infection site and division of labour in the redial stage (Poulin et al., 2019b; Presswell and Bennett, 2020; Viriyautsahakul et al., 2025). In this study, molecular techniques revealed that *G. serpentum* specimens recovered from the gallbladder, regardless of the presence or absence of gonads, as well as those from the small intestine, are conspecific. Although the reasons behind the absence of gonads in some individuals of *G. serpentum* are unknown, this provides strong support for the necessity of incorporating genetic identification alongside morphological assessment when describing helminth species.

Additionally, the use of markers from both nuclear and mitochondrial DNA is recommended to ensure accurate species delimitation. This is exemplified by *G. bijirrii* and *G. acrochordi*, which show no sequence variation in the nuclear 28S and ITS2 markers, yet display some differences in *COI* sequences (Achatz et al., 2024). However, these mitochondrial differences may reflect intraspecific variation rather than confirming them as distinct species (Chan et al., 2021), particularly because these taxa occur in geographically distant populations in Australia (Pérez-Ponce et al., 2016; Sromek et al., 2023). Therefore, although they differ morphologically, their identity as separate species requires further investigation.

Although the phylogenetic analysis revealed that the individuals without gonads are merely morphological variants of *G. serpentum* rather than a separate species, the reasons behind the absence of gonads remain unclear. The presence of eggs within the uterus of all 3 gonad-less individuals (Figure 1E) and in the individual with only one testis (Figure 1D) suggests that these trematodes initially developed gonads, which subsequently regressed through an unknown mechanism driven by unidentified biological factors. One possible explanation is intraspecific competition, as high infection intensity may lead to overcrowding and reduced body condition, which has been previously observed in smaller average body size of the parasites from heavily infected hosts (Saldanha et al., 2009; Viriyautsahakul et al., 2025). However, the no-gonad parasites in this study were recovered from hosts with low to moderate infection intensities (*n* = 1–33), exhibited average body sizes compared to other individuals. Thus, the discovery of complete gonad disappearance in *G. serpentum* represents an intriguing phenomenon and warrants further investigation in future studies.

It is notable that the variation involving the absence of gonads has only been reported in the population infecting *E. enhydris*, which was previously described as *G. burmanicus* (Chatterji, 1940), while the no-gonad individuals have never been reported in the *G. serpentum* infecting other snakes (Simha, 1958; Frank, 1966). This may suggest some degree of host incompatibility. However, this also seems unlikely, as such individuals were found in only 3 out of 119 snakes examined, and the overall average egg numbers of the parasites (4 ± 1) from this study were greater than the other population (i.e. 1–3; Chatterji, 1940), suggesting high fecundity of parasites.

The discovery of *G. serpentum* in southern Thailand has extended its known distribution across Southeast Asia and India. Previously, *G. serpentum* was primarily reported from the checkered keelback (*F. piscator*), where it infects the small intestine. However, its occurrence in the gallbladder of tentacled snakes and in both the gallbladder and small intestine of rainbow water snakes (*E. enhydris*) suggests that this parasite is a generalist capable of infecting multiple, distantly related snake species (Mehra, 1947; Frank, 1966). This pattern resembles that of *Encyclometra bungara*, another trematode found in the same *E. enhydris* population, which is widespread across India and Southeast Asia and infects multiple organs in four different snake species (Chan et al., 2024). *Encyclometra bungara* also exhibits extensive morphological plasticity across its range, similar to that observed in *G. serpentum* in this study. Given that both *E. enhydris* and *F. piscator* are widespread and adaptable throughout South and Southeast Asia, it is unsurprising that *G. serpentum* also shows high plasticity to adapt to different host populations and geographical areas (Karns et al., 2010; Devkota et al., 2020). Morphological variations, such as differences in body length, may reflect this plasticity, although underlying genetic differences are also possible (Blasco-Costa et al., 2016; Chan et al., 2024). Broad-scale genetic analyses are needed to assess genetic differentiation among *G. serpentum* populations and to delineate intraspecific variation from potential interspecific divergence (Blasco-Costa et al., 2016).

*Gogatea serpentum* is the fourth species of helminth recovered from *E. enhydris* in the Nakhon Si Thammarat locality, alongside *E. bungara, Tanqua siamensis*, and *Paratestophis gelicolus*, each of which parasitizes different target organs within the snake (Chan et al., 2024; Charoennitiwat et al., 2024, 2025). Co-infection by multiple helminths is common in snakes, as their carnivorous diet and high trophic level increase their likelihood of acquiring trophically transmitted helminths through predation (Santoro et al., 2012; Davis et al., 2016; Oliveira et al., 2024; Kirillov et al., 2025). In addition, snakes with semi-aquatic lifestyles face an even higher risk of infection because their primary prey items, such as frogs and fish, serve as important intermediate hosts for many helminths (Lagrue et al., 2011; Kirillov et al., 2025). Individual semi-aquatic snakes can host as many as 26 different helminth species (Oliveira et al., 2024; Kirillov et al., 2025). These findings suggest that *E. enhydris*, as an aquatic snake, plays an important role as a definitive host for a diverse community of helminths within its locality. Future studies on the parasite assemblages of its potential prey items may help elucidate the transmission routes of these helminths as well as predator–prey links within the local food web (Ratnarathorn et al., 2025).

In conclusion, this study reports a new population of *G. serpentum* (Gogate, 1932) from the rainbow water snake, *E. enhydris*, in southern Thailand. Morphological variation was observed among the parasites, most notably the absence of testes in some individuals, which corresponds to the description of *G. burmanicus* (Chatterji, 1940). However, molecular analyses showed that all specimens, with or without gonads, were completely identical across 3 markers, confirming the conspecificity of *G. serpentum* and *G. burmanicus*. Accordingly, *G. burmanicus* syn. n. should be regarded as a synonym of *G. serpentum*, despite the marked morphological differences. This finding underscores the importance of incorporating molecular analyses across multiple gene regions alongside traditional morphology, as intraspecific variation is likely widespread among helminths. The mechanisms underlying gonadal loss remain unclear, warranting further investigation. Finally, this species represents the fourth helminth and third trematode reported from *E. enhydris*, highlighting the significant role of aquatic snakes as definitive hosts of the helminths and pointing to the need for studies on their prey to better understand local parasite transmission routes and food web dynamics.

## Supporting information

10.1017/S0031182025101054.sm001 Viriyautsahakul et al. supplementary material Viriyautsahakul et al. supplementary material

## Data Availability

The data that support the findings of this study are available from the first and corresponding authors upon reasonable request.

## Supplementary material

The supplementary material for this article can be found at https://doi.org/10.1017/S0031182025101054.

## Table 1 Long description

The table compares morphological features and measurements of Gogatea serpentum and Gogatea burmanicus, focusing on body length, forebody length, and organ dimensions. Gogatea serpentum, studied in Thailand, exhibits a wider range of body lengths and more variability in features like prosoma and opisthosoma dimensions compared to Gogatea burmanicus from Myanmar. Key differences include the presence of gonads in some Gogatea serpentum specimens and their absence in Gogatea burmanicus. The table also highlights variations in host species and infection sites, with Gogatea serpentum primarily infecting the gallbladder of Enhydris enhydris, while Gogatea burmanicus is found in the intestine of Erpeton tentaculatum. These differences suggest ecological and biological diversity between the species and across different geographical locations.

Navigate back to Table 1.

## Figure 1 Long description

The image A shows the entire body of a mature Gogatea serpentum specimen with complete gonad, ventral view. It includes labels for anatomical features such as oral sucker, caeca bifurcation, oesophagus, ovary, eggs, anterior testis, posterior testis, metraterm, cirrus sac, genital atrium, holdfast organ, prosoma and ophistosoma. The image B shows the anterior region of a specimen, ventral view, with labels for pharynx, caecum, ventral sucker and vitelline follicle. The image C shows the entire body of an immature specimen, ventral view, without specific labels. The image D shows the entire body of a mature specimen with incomplete gonad, ventral view, highlighting one testis. The image E shows the entire body of a mature specimen without gonad, ventral view, without specific labels.

Navigate back to Figure 1.

## Figure 2 Long description

Illustration A shows the entire body of a mature specimen with complete gonads, ventral view. Labels include oral sucker, pharynx, oesophagus, ventral sucker, eggs, anterior testis, ovary and posterior testis. Illustration B depicts the entire body of a mature specimen lacking gonads, ventral view, with similar labels. Illustration C shows the holdfast organ, ventral view, labeled with vitelline follicle. Illustration D presents the posterior region of a mature specimen with complete gonads, ventral view, labeled with ovary, anterior testis, metraterm, posterior testis and cirrus sac. Each illustration includes a scale bar indicating 100 micrometres.

Navigate back to Figure 2.

## Figure 3 Long description

The image A shows the entire body of Gogatea serpentum with a large protruded holdfast organ, ventral view. The image B shows the anterior region with densely distributed dot-like tegumental spines, dorsal view. The image C shows the oral sucker in the anterior region, ventral view. The image D shows a large protruded holdfast organ with a small ventral sucker anteriorly, indicated by a white arrow, ventral view. The image E shows the genital pore at the apical end, ventral view. The image F shows densely distributed tegumental spines on the anterior body. The image G shows sparsely distributed tegumental spines on the posterior body. The image H shows a magnified view of a tegumental spine.

Navigate back to Figure 3.

## Figure 4 Long description

A scatter plot illustrating principal component analysis with two axes labeled PC1 and PC2. The x-axis is labeled PC1 with a percentage of 52.147 percent and the y-axis is labeled PC2 with a percentage of 15.178 percent. Black dots represent gonad-bearing specimens, while red triangles represent gonad-less specimens. The plot is enclosed within a polygonal boundary, showing the distribution of specimens across the axes.

Navigate back to Figure 4.

## Figure 5 Long description

The image A shows a phylogenetic tree based on the 28S rRNA marker. It includes various Gogatea species, with Gogatea serpentum specimens highlighted in red. The tree shows branch lengths and bootstrap values, with Cyathocotyle prussica as the outgroup. The image B shows a phylogenetic tree based on the ITS2 marker, featuring Gogatea species and highlighted Gogatea serpentum specimens. Cyathocotyle prussica serves as the outgroup. The image C shows a phylogenetic tree based on another genetic marker, displaying Gogatea species with Gogatea serpentum highlighted. Cyathocotyle prussica is the outgroup. The image D shows a phylogenetic tree using concatenated sequences from all three markers, illustrating relationships among Gogatea species with Gogatea serpentum highlighted. Cyathocotyle prussica is the outgroup. Each tree includes branch lengths and bootstrap values, indicating the number of substitutions per site.

Navigate back to Figure 5.
