# Supplementary material for: Hidden in morphology, revealed by molecular genetics: synonymization of Gogatea burmanicus (Chatterji, 1940) with Gogatea serpentum (Gogate, 1932) (Digenea: Cyathocotylidae)
Source: Parasitology. 2025 Nov 6;153(4):433–44. doi: 10.1017/S0031182025101054 (PMC13244229; doi:10.1017/S0031182025101054)
Supplement: Viriyautsahakul et al. supplementary material [file S0031182025101054sup001.zip › S0031182025101054sup001/Table S4_Supplement.docx]

**Table S4:** Specific primers, thermal cycling conditions, and PCR amplification details for the molecular investigation used in this study.

| **Gene** | **Primer** | **Primer sequence** | **Length (bp)** | **Denaturation** | **Extension** | **Reference** |
| --- | --- | --- | --- | --- | --- | --- |
| 28S rRNA | Digl2 | 5′-AAG CAT ATC ACT AAG CGG–3′ | 1,200  (partial) | 94°C_3 min, 34 cycles of 94°C_45 sec, 54°C_45 sec 72°C_2 min | 72°C_10 min | Curran *et al*., 2011 |
|  | 1500R | 5′-GCT ATC CTG AGG GAA ACT TCG-3′ |  |  |  |  |
| ITS2 | 5.8SF-Echinochasmidae | 5′-CTG CTT TGA ACA TCG ACA TC-3′ | 995  (partial) | 94°C_5 min, 34 cycles of 95°C_30 sec, 55°C_30 sec, 72°C_2 min | 72°C_5 min | Besprozvannykh *et al*., 2018 |
|  | 28S4R | 5′-TAT TTA GCC TTG GAT GGA GTT TAC C-3′ |  |  |  |  |
| *COI* | Dice1F | 5′-AAT AAC CCT CAC TAA ATT WCN TTR GAT CAT AAG-3′ | 714  (partial) | 94°C_2 min, 3 cycles of 94°C_40 sec, 51°C_40 sec, 72°C_1 min, 5 ‘touchdown’ cycle of 94°C_40 sec, 50°C to 46°C _40 sec (dropping 1°C per cycle, 72°C_1 min; 35 cycles of 94°C_40 sec, 45°C_40 sec, 72°C_1 min | 72°C_5 min | Van Steenkiste *et al.*, 2015 |
|  | Dice14R | 5′-TAA TAC GAC TCA CTA TAC CHA CMR TAA ACA TAT GAT G -3′ |  |  |  |  |
