# Supplementary material for: Hidden in morphology, revealed by molecular genetics: synonymization of Gogatea burmanicus (Chatterji, 1940) with Gogatea serpentum (Gogate, 1932) (Digenea: Cyathocotylidae)
Source: Parasitology. 2025 Nov 6;153(4):433–44. doi: 10.1017/S0031182025101054 (PMC13244229; doi:10.1017/S0031182025101054)
Supplement: Viriyautsahakul et al. supplementary material [file S0031182025101054sup001.zip › S0031182025101054sup001/Table S5_Supplement.docx]

**Table S5:** GenBank sequences of all available *Gogatea* species and the designated outgroup used for phylogenetic analysis.

| **Species** | ***Genetic marker*** | | |
| --- | --- | --- | --- |
|  | **28S** | **ITS2** | ***COI*** |
| *Gogatea bijirrii* | PP992060 | PP992062 | PP991451 |
| *Gogatea bijirrii* | PP992061 | PP992063 | PP991450 |
| *Gogatea bijirrii* | - | - | PP991452 |
| *Gogatea acrochordi* | PP992055 | PP992056 | PP991445 |
| *Gogatea acrochordi* | PP992053 | PP992057 | PP991446 |
| *Gogatea acrochordi* | PP992067 | - | PP991444 |
| *Gogatea anacetabulata* | PP992058 | PP992058 | PP991448 |
| *Gogatea anacetabulata* | PP992059 | PP992059 | PP991447 |
| *Gogatea mehri* | PP992064 | PP992065 | PP991454 |
| *Gogatea mehri* | MK650441 | PP992064 | PP991453 |
| *Gogatea serpentum* SN172 | This study | This study | This study |
| *Gogatea serpentum* SN117 | This study | This study | This study |
| *Gogatea serpentum* (*G. burmanicus* syn. n.) | This study | This study | This study |
| *Gogatea* sp. | MK650442 | PP992066 | PP991455 |
| *Cyathocotyle prussica* (outgroup) | MH521249 | PP849714 | MH536510 |
